# *Novosphingobium* and Its Potential Role in Chronic Obstructive Pulmonary Diseases: Insights from Microbiome Studies

**DOI:** 10.1371/journal.pone.0111150

**Published:** 2014-10-23

**Authors:** Alleluiah Rutebemberwa, Mark J. Stevens, Mario J. Perez, Lynelle P. Smith, Linda Sanders, Gregory Cosgrove, Charles E. Robertson, Rubin M. Tuder, J. Kirk Harris

**Affiliations:** 1 University of Colorado School of Medicine, Program in Translational Lung Research, Division of Pulmonary Sciences and Critical Care Medicine, Anschutz Medical Campus, Aurora, Colorado, United States of America; 2 University of Colorado - School of Medicine, Department of Pediatrics, Pulmonary Medicine, Mucosal and Vaccine Research Colorado, Microbiome Research Colorado, Children’s Hospital Colorado, Aurora, Colorado, United States of America; 3 National Jewish Health, Department of Medicine and University of Colorado School of Medicine, Division of Pulmonary Sciences and Critical Care Medicine, Anschutz Medical Campus, Aurora, Colorado, United States of America; 4 Department of Molecular, Cellular and Developmental Biology, University of Colorado, Boulder, Colorado, United States of America; Queens University Belfast, Ireland

## Abstract

Bacterial infection of lung airways underlies some of the main complications of COPD, significantly impacting disease progression and outcome. Colonization by bacteria may further synergize, amplify, or trigger pathways of tissue damage started by cigarette smoke, contributing to the characteristic airway inflammation and alveolar destruction of COPD. We sought to elucidate the presence and types of lung bacterial populations in different stages of COPD, aimed at revealing important insights into the pathobiology of the disease. Sequencing of the bacterial small subunit ribosomal RNA gene in 55 well-characterized clinical lung samples, revealed the presence of *Novosphingobium* spp. (>2% abundance) in lungs of patients with GOLD 3-GOLD 4 COPD, cystic fibrosis and a subset of control individuals. *Novosphingobium*-specific quantitative PCR was concordant with the sequence data and high levels of *Novosphingobium* spp. were quantifiable in advanced COPD, but not from other disease stages. Using a mouse model of subacute lung injury due to inhalation of cigarette smoke, bronchoalveolar lavage neutrophil and macrophage counts were significantly higher in mice challenged intratracheally with *N. panipatense* compared to control mice (p<0.01). Frequencies of neutrophils and macrophages in lung tissue were increased in mice challenged with *N. panipatense* at room air compared to controls. However, we did not observe an interaction between *N. panipatense* and subacute cigarette smoke exposure in the mouse. In conclusion, *Novosphingobium* spp. are present in more severe COPD disease, and increase inflammation in a mouse model of smoke exposure.

## Introduction

Diverse microbial communities are found in each of the different environments of human body, where they interact with resident inflammatory cells in both health and disease [1]–[6]. Recently, studies carried out by multiple consortiums and individual investigations [1], [7], [8] have contributed significantly to our understanding about the human microbiome. The respiratory tract, like the gastrointestinal and skin systems, is continuously exposed to environmental agents; moreover, many pulmonary diseases lead to increased risk of infections and altered microbial communities [2], [3], [9], [10]. It is anticipated that the better understanding of the microbiome in healthy and diseased lungs may better inform how diseases are modified in their course by infection and provide novel means to implement disease-specific treatments [11].

Until the introduction of high throughput analysis of bacterial population using 16S ribosomal RNA amplification, the lungs of healthy individuals were thought to be sterile, while those of diseased patients were colonized with bacteria that were associated with disease, the advent of molecular techniques shows this not to be so [12], [13]. The lung is continuously challenged by inhaled particulates and microorganisms, and yet, healthy individuals rarely have bacteria isolated from their airways. In fact, there is a growing body of evidence of organisms, not identifiable by classical cultivation efforts, present within the human airway [14]–[17].

Bacterial infections of the lung airways and parenchyma play a central role in the progression, severity, and outcome of airway diseases, including chronic obstructive pulmonary disease (COPD) [9]. COPD is largely caused by cigarette smoking [18], affecting approximately 16 million Americans and will become the third most common cause of worldwide morbidity in the next few years [19]. Of particular importance in COPD are acute exacerbations caused by bacteria and viruses, contributing to morbidity, clinical deterioration, and mortality among patients with COPD [20], [21]. Acute exacerbations (AE) in COPD are an acute and sustained progression of the disease beyond normal day-to-day variations, requiring change in medication and/or hospitalization [22]. The average COPD patient has two episodes of acute exacerbations per year, with 10% requiring hospitalization that lasts on average approximately six days [23]. Bacteria account for 30–50% of overall AEs, while viruses are detected in 25% of cases [24].

Even patients with stable COPD (40%) have evidence of bacteria in their secretions, which are considered to represent colonization, often consisting of *Haemophilus influenzae*, *Moraxella catarrhalis*, *Streptococcus pneumoniae*, and *Pseudomonas aeruginosa* [20]. A low microbial load, i.e., 10^2^ to 10^3^ colony forming units (cfu)/ml is seen in 25% of stable patients while 10% of stable patients may present with high bacterial load [25]. Colonization is associated with heightened expression of markers of inflammation [26]; colonized patients have also worse overall health and increased expression of serum fibrinogen, as a marker of systemic inflammation, when compared with COPD patients without colonization [27], [28].

Sources of bacteria may also involve the environment and, in particular, processed tobacco and tobacco leaves. A recent study, based on SSU-rRNA, revealed multiple members of several phyla, including α-Proteobacteria, such as the genus *Novosphingobium*, in commercial cigarettes [29]. These findings complemented and expanded prior documentation of bacteria grown in standard cultures from processed cigarettes and cigarette filters, and of endotoxin in both cigarettes and samples of air in smoking environments [29]. This evidence underscores the need for high throughput approaches that may identify whether processed tobacco (as present in cigarettes) might also be a source of bacterial infection, colonization and exposure, as the result of cigarette smoke inhalation.

Since the interplay of lung infection by bacterial and/or viruses and lung inflammation may synergize, amplify, or trigger pathways of tissue damage started by cigarette smoke [22], , we sought to identify whether unique bacterial populations are present in COPD lungs and might affect lung inflammation [31]. These insights might have broader implications in the understanding of any potential involvement of bacterial populations in the course of cigarette smoke-induced inflammation, alveolar injury (cell death), and clinical exacerbations.

## Materials and Methods

### Human Samples

All human material was approved through the Colorado Combined Institutional Review Board. Lung samples included subjects who were non-normal smoker’s lungs, normal smoker’s lungs, lung samples from donors with no or minimal disease (GOLD 1/2), and lungs with moderate/severe disease (GOLD 3/4) were obtained from the Lung Tissue Repository Consortium of the National Heart Lung and Blood Institute (LTRC). Freshly frozen lung samples from subjects with advanced cystic fibrosis disease were evaluated as controls for the presence of bacteria were obtained from the Colorado Children’s Hospital; freshly frozen normal nonsmokers’ and smokers’ samples consisted from explanted lungs, not used for lung transplantation, were provided by the National Jewish Health Interstitial Lung Disease Program biorepository. The clinical characteristics of the adult study population are described in Table 1. Table 1 does not contain information on all samples included due to lack of demographic information from some tissues sources. A single sample was examined in triplicate during sample processing, and the inclusion of the replicate libraries did not affect the overall results.

**Table 1:** Demographic and clinical characteristics of the study population.

Group ID	Age	MeanAge	GOLDstage	Gender	Gender(M/F)	Ethnicity	SmokeCig?	Packyears	Meanpackyears	FEV1/FVC
Normal		48.5			3/2				24.4	
Lungs	47			M		na	Yes	2		
	65			F		na	Yes	40		na
	na			M		na	No	0		na
	44			F		na	Yes	60		na
	38			M		na	Yes	20		na
G0		55			1/3				36.3	0.76
	64		0	M		1	Yes	96		na
	55		0	F		1	Yes	6		0.68
	49		0	F		1	Yes	7		0.82
	52		0	F		2	No	0		0.78
G1		61.5			0/2				35	0.64
	65		1	M		1	Yes	50		0.60
	58		1	M		1	Yes	20		0.67
G2		56.2			2/3				47.6	0.58
	60		2	F		1	Yes	48		na
	58		2	F		1	Yes	45		0.69
	59		2	M		1	Yes	75		0.66
	58		2	F		1	Yes	25		0.50
	46		2	M		1	Yes	45		0.47
G3	56	56	3	F	1/0	1	Yes	45	45	0.45
G4		56.8			8/10				47.4	0.31
	58		4	M		1	Yes	20		na
	63		4	M		1	Yes	84		0.17
	51		4	M		1	Yes	116		0.33
	48		4	F		1	Yes	28		0.50
	55		4	F		1	Yes	37		0.21
	49		4	M		1	Yes	36		0.27
	65		4	M		1	Yes	45		0.32
	63		4	F		1	Yes	120		0.26
	52		4	F		1	Yes	1		0.57
	64		4	M		1	Yes	113		0.36
	45		4	M		1	Yes	20		0.40
	60		4	M		1	Yes	20		0.23
	55		4	F		1	Yes	20		0.21
	63		4	F		1	Yes	44		0.24
	60		4	F		1	Yes	9		0.42
	64		4	F		1	Yes	40		0.29
	57		4	F		1	Yes	30		0.25
	50		4	F		1	Yes	70		0.20

Abbreviations: G = GOLD stage; na = not available; F = Female, M = Male; 1 = Not Hispanic, 2 = Hispanic; FEV1 = forced expiratory volume in 1 second, FVC = Forced Vital Capacity.

### DNA Extraction and Reagent Controls

Prior to embedding tissue and extracting DNA for subsequent sequence analysis, reagents were tested for the presence of bacterial DNA. Two lung samples taken from patients with COPD (GOLD stage 1 and GOLD stage 4, respectively) were processed to check for potential contamination. Small fragments were taken from each sample and embedded in optimal cutting temperature (OCT) embedding media, flash frozen in liquid nitrogen, and cut into 5 µm frozen sections. Controls included frozen sections of OCT media devoid of tissue fragments, prepared with the same equipment and consumables used for tissues. All-bacteria qPCR analysis was performed, and based on the presence of DNA in the OCT liquid alone (387 copies) versus COPD GOLD stage 1 (713 copies), versus GOLD stage 4 (1645 copies) compared to 35 copies in the qPCR (reagent) blanks, subsequent reagents underwent UV treatment before embedding in order to reduce background.

All DNA extractions were performed using the Qiagen EZ1 Advanced Extraction Platform with the Tissue Kit and Bacterial Card as per manufacturer’s instructions. Elution volume of 100 µl was selected and elution tubes with DNA extract were stored at −20°C. All extraction reagents were negative for bacterial DNA contamination.

### Bacterial Load Assay

Total ribosomal RNA gene copy number was measured using a quantitative PCR (qPCR) [32]. The primer set used included the forward primer, 5-TCCTACGGGAGGCAGCAGT-3, the reverse primer, 5-GGACTACCAGGGTATCTAATCCTGTT-3 and the probe, (6-FAM)-5-CGTATTACCGCGGCTGCTGGCAC-3-(TAMRA). The reaction conditions for amplification of DNA was 95°C for 7 minutes and 40 cycles of 95°C for 15 seconds and 60°C for 1 minute. Cloned ribosomal RNA gene DNA from the bacterium *Prevotella melaninogenica* was used as the standard, ranging in dilution from 10^2^ to 10^8^ copies on each plate.

### DNA Sequencing

Pyrosequencing of barcoded 16S rRNA gene amplicons targeting the V1-2 region were used to determine the bacterial communities present in each sample as previously described [33], using forward primer 5′-GCCTTGCCAGCCCGCTCAGTCAGAGTTTGATCCTGGCTCAG-3′ and the reverse primer 5′-GCCTCCCTCGCGCCATCAGNNNNNNNNCATGCTGCCTCCCGTAGGAGT-3′. DNA extracts were amplified in triplicate along with a negative PCR control. Any sample where the negative control was positive was repeated. Amplicons were normalized using the SequalPrep Plate (Invitrogen) as previously described [34] mixed in equal amounts and sequenced per manufacturer’s instructions using the Roche 454 pyrosequencing platform. Post sequencing informatics were performed as previously described [35]. This included basic quality assessment (sequences <200 nucleotides [nt] in length, >1 nt ambiguity, best read with quality > = 20 over a 10 nt window) using Bartab [36], chimera detection using ChimeraSlayer [37]. Sequences that did not pass quality checks were removed from the analysis. Taxonomic classification was performed using RDP-Classifier [38], and unclassified sequences were also removed from the dataset to exclude non-bacterial sequences. Relative abundance was utilized to normalize the difference in sequence count between samples, and all graphics were produced using Explicet (www.explicet.org) [39]. We utilized BLAST to further investigate the *Novosphingobium* sequences, and identified *N. panipatense* as the closest isolate sequence (>99% sequence identity over >95% sequence length) to those obtained by pyrosequencing. DNA sequence data has been deposited in NCBI under the accession SRP039563.

### Bacterial Strains, Growth and Quantification

*Novosphingobium panipatense* (Deutsche Sammlung von Mikroorganismen und Zellkulturen GmbH, DSMZ, Germany) was grown in Trypticase Soy broth (Becton Dickinson, MD, USA) supplemented with 200 µg/ml Streptomycin at 29°C for 48 hours with shaking for all experiments. Bacterial enumeration was performed by generating ten-fold dilutions of bacterial growth culture in PBS and culturing 100 µL on Trypticase Soy Agar plates (Becton Dickinson, MD, USA) supplemented with 200 ug/ml Streptomycin (Sigma, USA) at 29°C for 48 hours. Bacterial enumeration was also evaluated in mice using 10–100 µL of mouse BAL cell pellets resuspended in PBS cultured on Trypticase Soy Agar plates at 29°C for 48 hours.

### Quantification of *Novosphingobium* spp. using specific real-time PCR assay

DNA extracted from cultures of *Novosphingobium panipatense* was used to generate a genome copy number standard curve by plotting a linear regression curve with the mean CT values on Y-axis and logarithm of the copy number on X-axis. Amplification efficiencies, coefficients of determination (R2) and curve slopes were auto-calculated by the Mastercycler EP Realplex (Eppendorf) analysis program. To determine PCR sensitivity through serial log dilutions within a range of 10^2^ to 10^8^ bacterial genomic molecules. The bacterial dilutions were subjected to real-time PCR as described earlier.

### Quantification of *Novosphingobium* spp. in Lung Tissue Samples

The extracted DNA was amplified using pan-bacterial primers for the first round PCR. Primers were synthesized commercially (Integrated DNA Technologies, IA). The 27F forward primer 5′-GCCTTGCCAGCCCGCTCAGTCAGAGTTTGATCCTGGCTCAG-3′ and 805R reverse primer 5′-GACTACCAGGGTATCTAAT-3′. Reactions were initiated at 95°C for 10 min, followed by 27 cycles of 94°C for 15 s, 60°C for 30 s, and 72°C for 30 s. Subsequent amplification was performed using *Novosphingobium* spp. specific primers, forward primer 5′-TCCGAGTGTAGAGGTGAAAT-3′ which recognizes several sequences in the α-Proteobacteria and reverse primer 5′-CGTCAATACTTGTCCAGTCA-3′ which has high specificity for *Novosphingobium*. Each quantitative real-time PCR (qPCR) reaction contained 10 µl of “SYBR Green Master Mix” (Applied Biosystems Inc), 0.2 µl each of the *Novosphingobium* forward and reverse primers at 3 µM each, 5.6 µl of DNA free water (Fisher Scientific, CA), and 4 µl of primary PCR product. Assays were performed using the Mastercycler EP Realplex (Eppendorf, USA). Reactions were initiated at 95°C for 10 min, followed by 35 cycles of denaturation at 95°C for 15 s, annealing at 61°C for 15 s. Dissociation or melting reaction, to determine the specificity of the products on the basis of the melting temperature, consisted of incubation at 95°C for 15 s, annealing at 60°C for 20 s, followed by slowly increasing the temperature to 95°C over 20 min. A single peak was obtained with the absence of any extra peak(s). The detection limit of the PCR assay (standard curve method) was less than100 genome equivalents of *Novosphingobium panipatense* per reaction. The electrophoresis run for the PCR products indicated specificity of a product band of ∼100 base pairs corresponding to the *Novosphingobium panipatense* gene product (data not shown).

Initial attempts to perform *Novosphingobium* spp. specific qPCR resulted in the amplification of multiple products due to the presence of both the pan-bacterial primers that may have carried over from the first round of PCR amplification, and the *Novosphingobium* spp. primers used for the second round of amplification. In order to eliminate the amplification of non-specific products in subsequent steps, the first round PCR product underwent a cleanup treatment with EXOSAP-IT as per manufacturer’s instructions with minor modifications (samples were treated with EXOSAP-IT at 37°C overnight (for 15–18 hours) followed by 15 minutes at 80°C to inactivate the reaction). This step removed unwanted dNTPs and primers that remained from the first round PCR product before proceeding to perform nested PCR or qPCR. Melting Curves confirmed that the PCR products amplified after performing EXOSAP-IT cleanup treatment resulted in PCR product that demonstrated melting curves similar to those seen with the *Novosphingobium* DSZM stocks (80°C).

### Animal Studies

Multiple studies have demonstrated differences in susceptibilities for the development of smoking-induced emphysema between strains of mice [40]–[42]. The C57BL/6 mouse model was chosen for this study because in prior studies, it has been shown to development acute and chronic injury due to cigarette smoke, including lung inflammation, cell death, oxidative stress, and emphysema [42], [43]. Mouse studies were approved by the Institutional Animal Care and Use Committee at the University of Colorado Denver. The C57BL/6 mice (male, 6–8 weeks old) used were purchased from The Jackson Laboratory (The Jackson Laboratory, California, USA). A total of ten mice were used per experimental/test group.

### Intratracheal inoculation and Bacterial challenge

To demonstrate the effects of *Novosphingobium* spp. infection in lung compartments, such as airways and alveolated tissue, C57BL/6 mice in the experimental group were challenged once every seven days with 50 µl of PBS containing 5×107 CFU total of Novosphingobium panipatense for a total of six weeks (i.e. six bacterial intratracheal challenges given at a rate of one challenge per week), while control mice were subjected to challenges with 50 µl sterile PBS, instilled intratracheally. Mice were then exposed to room air conditions for six weeks or were exposed for six weeks to cigarette smoke generated by the TE-10 Smoking Machine (Teague Enterprises) and 3R4F reference cigarettes (University of Kentucky, Tobacco Research Institute, Lexington), as previously described [43], [44]. In brief, mice were exposed to one smoking exposure a day for 6 hours, 5 days a week for 6 weeks cigarette smoke exposure was carried out by burning 3R4F reference cigarettes (2.45 mg nicotine per cigarette) in the smoking machine. Each smoldering cigarette was puffed for 2 seconds, once every minute for a total of 8 puffs, at a flow rate of 1.05 l/min, to provide a standard puff of 35 cm^3^. The smoke machine was adjusted to produce a mixture of side stream smoke (89%) and mainstream smoke (11%) by burning between 3–6 cigarettes at one time. This smoke was then mixed with room air and transferred to the smoking chamber at a flow rate of 25 liters per minute. Chamber atmosphere was monitored for total suspended particulates and carbon monoxide, with concentrations of 110 mg/m^3^ and 350 ppm, respectively. Temperature and humidity in the chamber were the same as in the room housing the cigarette smoking machine.

### Bronchoalveolar Lavage

Mice were sacrificed and the thoracic cavity opened to expose the lungs. After cannulating the trachea, the bronchoalveolar lavage (BAL) fluid was collected by instilling 1 ml ice-cold Phosphate Buffered Saline into the lung with subsequent aspiration for collection, this was repeated three times. Total viable cell counts were determined in a hemocytometer using trypan blue exclusion. Differential counts of neutrophils and macrophages were determined on cytospin smears of BAL samples. In brief, 150 µL of cell suspension was centrifuged in a Shandon Single Cytofunnel (Thermo Scientific, USA) at 650 rpm for 3 minutes. The slide produced was air dried, fixed and stained using 3 Hema 3 step stain set (Fisher Scientific, USA). The slides were rinsed in water twice and allowed to dry, then cover glasses were applied with mounting medium (Fisher Scientific) and cells were scored. Results were expressed as total cell number/mL. BAL fluid collection was then centrifuged (3 min; 1000 rpm). Supernatant (0.5–1.0 mL) was harvested and flash frozen in liquid nitrogen prior to storage at −80°C. Cell pellets were resuspended in the residual PBS (0.5–1.0 mL) of which, up to 200 µL was stored at 4°C and used for subsequent bacterial cultures. The remaining cell pellet was flash frozen in liquid nitrogen prior to storage at −80°C.

### Lung Tissue Harvesting

A cannula was inserted into the trachea and lungs were flushed by perfusing 20 ml of PBS through the right ventricle. A pre-warmed solution of low melting point agarose (1% agarose in PBS) was introduced into the left lung under constant pressure (20 cm H_2_O), transferred into a plastic fixation cassette and placed in 10% formalin overnight, prior to being paraffin embedded. The superior and inferior lobes of the right lung were flash frozen in liquid nitrogen. The middle lobe of the right lung was transferred into 3 ml conical tube, and kept at 4°C for 5–20 min prior to being processed. The larger lobe of the right lung was placed in 60 mm tissue culture dishes dissected and minced into 1–2 mm diameter fragments containing pre-warmed dissociation solution. The lung tissue dissociation solution contained Dispase solution (0.6 U/mL; Life Technologies), DNase I (60 U/ml), and collagenase I (0.3 U/mL; Gibco Invitrogen). Lung fragments were disintegrated in this solution (30–60 min; 37°C) until a complete cell suspension was obtained. Cell suspension was filtered through a 40 µm cell strainer (Fisher Scientific, USA), followed by centrifugation (10 min, 1000 rpm). Cell pellets were washed twice with PBS/2% FBS (10 min, 1000 rpm) and resuspended in 0.5 mL PBS/2% FBS and used as needed.

### DNA extraction from Mouse Lung Tissue Samples

DNA extraction and quantification of *Novosphingobium* spp. on flash frozen mouse lung tissue samples was performed as described above.

### Quantification of *Novosphingobium* spp. in Mouse Lung Tissue Samples

Quantification of *Novosphingobium* spp. on DNA extracted from mouse lung tissue samples was performed as described above.

### Fluorescence-activated cell sorting analysis

Following lung tissue dissociation described above, single cell suspensions were first stained with the monoclonal antibodies Siglec F –PE, CD11c -PerCPCy5.5, Ly6G – APC, F4/80- e450 and CD11b - AF700 for 30 minutes in the dark on ice. Cells were then washed with cold PBS. After the cells were washed, they were resuspended in 2% paraformaldehyde and kept at 4°C prior to subsequent flow cytometry analysis. For each mouse experimental group, 50,000–100,000 events collected on an LSRII flow cytometer and analyzed for the presence of neutrophils (Ly6G+CD11b+), resident macrophages (CD11c+Siglec F+) and recruited macrophages (CD11b+Siglec F−).

### Statistical Analysis

Statistical analysis was performed using unpaired Student t test, ANOVA, or Kruskal-Wallis one-way ANOVA on ranks. Statistical difference was accepted at *P<*0.05.

## Results

### Patient Cohort

The clinical characteristics of 35 adult subjects from whom tissue samples were obtained are detailed in Table 1. Thirty tissue samples were from patients at risk of or diagnosed with COPD were studied, including GOLD 0 (n = 4), GOLD 1 (n = 2), GOLD 2 (n = 5), GOLD 3 (n = 1), and GOLD 4 (n = 18). The cohort was comprised of 16 males and 19 females. The mean age was 55.7 years (range 38–65 years, SD = ±7.2); 95% (33/35) subjects had a history of cigarette smoking. Subjects with moderate to late stage disease (GOLD 2–4) had an increased number of mean pack-year history compared to subjects with early stage disease (GOLD 0–1). These samples were compared with explanted lungs of children with cystic fibrosis, often harboring a rich microbial population [2]. Moreover, two colonic samples with necrotizing enterocolitis samples as positive controls for the presence of bacteria.

### Observed abundances of bacterial taxa in human lung tissues of diseased patients and of control subjects

**Figure 1:**
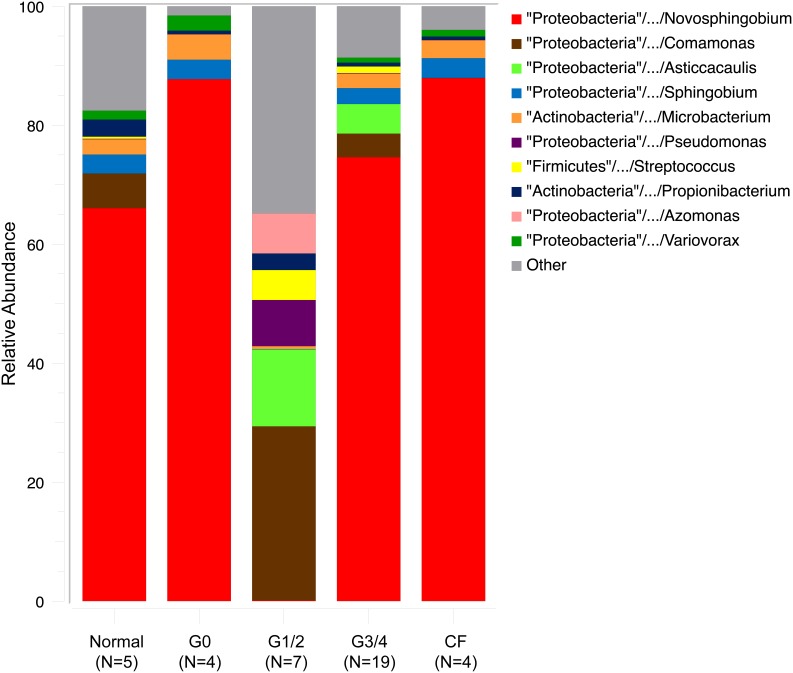
The top ten observed taxonomic lines based on total relative abundance across all groups, cumulative distributions are shown as the proportion of sequences observed per patient group.

Bacteria present in DNA extracted from fresh frozen human lung specimens obtained from control nonsmokers, smokers and patients with GOLD stages of 1–4 of severity of COPD and cystic fibrosis patients, were identified by phylogenetic analysis of amplified rDNA sequences by PCR with pan-bacterial primers. Figure 1 shows the top ten observed taxonomy abundances, cumulative distributions are shown as the proportion of sequences observed per patient group. The taxonomic groups of highest abundance (>3%) in the control ‘normal’ group were *Novosphingobium* (66%), *Comamonas* (6%), *Sphingobium* (3%) *Propionibacterium* (3%) and *Microbacterium* (3%); in the COPD GOLD 0 group, *Novosphingobium* (88%), *Microbacterium* (4%), and *Sphingobium* (3%); in the combined COPD GOLD 1/2 groups, *Comamonas* (29%), *Asticcacaulis* (13%), *Pseudomonas* (8%) and *Streptococcus* (5%); in the combined COPD GOLD 3/4 groups were *Novosphingobium* (59%) and *Streptophyta* (5%); and, finally, in patients with Cystic Fibrosis, *Novosphingobium* (39%), *Streptococcus* (8%), *Asticcacaulis* (8%) and *Acinetobacter* (4%, Figure 1).

### Quantification of *Novosphingobium* in human lung tissues of COPD patients and controls

Of the above list of bacteria identified in lungs, we focused in particular in *Novosphingobium* spp. Though considered oil organisms, Sphingomonadaceae (the family to which *Novosphingobium* spp. belongs) have been found to cause, albeit infrequently, lung disease in immunosuppressed individuals [45], [46]; of interest is the finding that the sphingomonas are also present in tobacco leaves, metabolizing polyhydrocarbons as nutrients [47]. Furthermore, *N. aromaticivorans* has been mechanistically linked to the autoimmune disease primary biliary cirrhosis [46], a paradigm similar to the proposed autoimmune nature of COPD [31]. Subsequent to performing extensive sequencing for microbiota in the human normal and diseased lung samples, we proceeded to quantify levels of *Novosphingobium* spp. in a subset of DNA extracted from the lung patient samples used for the sequencing studies.

**Figure 2:**
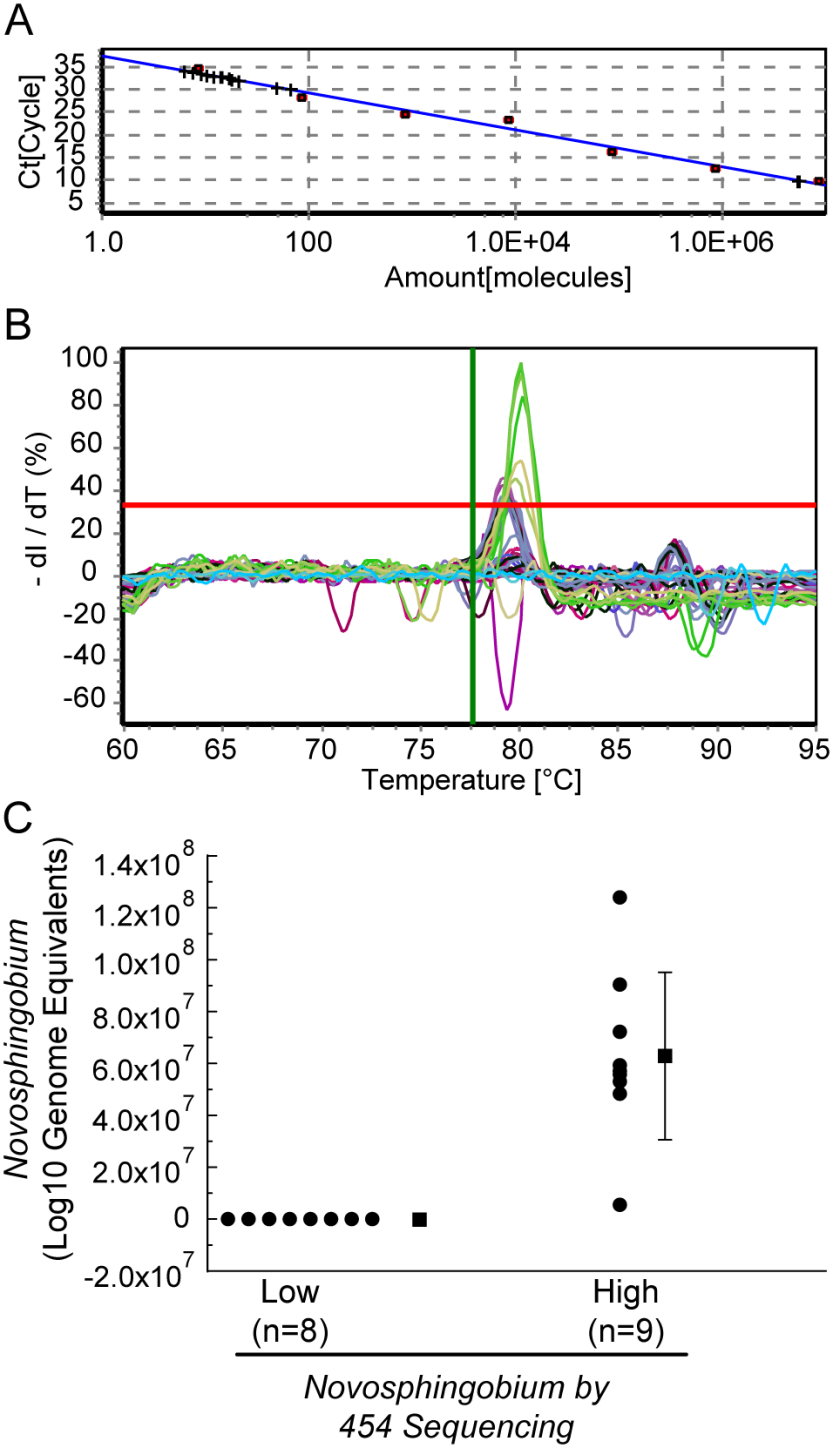
Quantification of *Novosphingobium*. (A) Standard curve generated using *Novosphingobium panipatense* and (B) confirmation by melting curve analysis of quantification of levels of *Novosphingobium* spp. (C) human lung specimens with low or undetectable levels of bacteria by sequencing (n = 8) and specimens with detectable levels of the bacteria (n = 9) by sequencing. An optimized two-step qPCR amplification protocol was used to measure levels of *Novosphingobium* spp. as described in Methods.

To calculate lung *Novosphingobium*-specific bacterial load, we developed a *Novosphingobium*-specific quantitative PCR strategy. First, we generated a standard curve using known genome equivalents of *Novosphingobium panipatense* DNA (Figure 2A), and confirmed the specificity based on melting curves (Figure 2B). We then quantified levels of *Novosphingobium* spp. in eight patient samples for which levels of the bacteria were low or undetectable (<2% abundance) and nine patient samples for which levels of the bacteria were >2% abundance. Using the optimized specific PCR amplification protocol, *Novosphingobium* was detectable (>100 genome equivalents) in all lung samples for which levels of the bacteria were >2% abundance (of all sequencing DNA reads). *Novosphingobium* was undetectable in lung samples for which levels of the bacteria were <2% abundance by sequencing (Figure 2C). Quantification of levels of *Novosphingobium* spp. by qPCR corroborated levels of *Novosphingobium* spp. that were observed by DNA sequencing.

### The role of *Novosphingobium* spp. in smoke induced lung injury in mice

**Figure 3:**
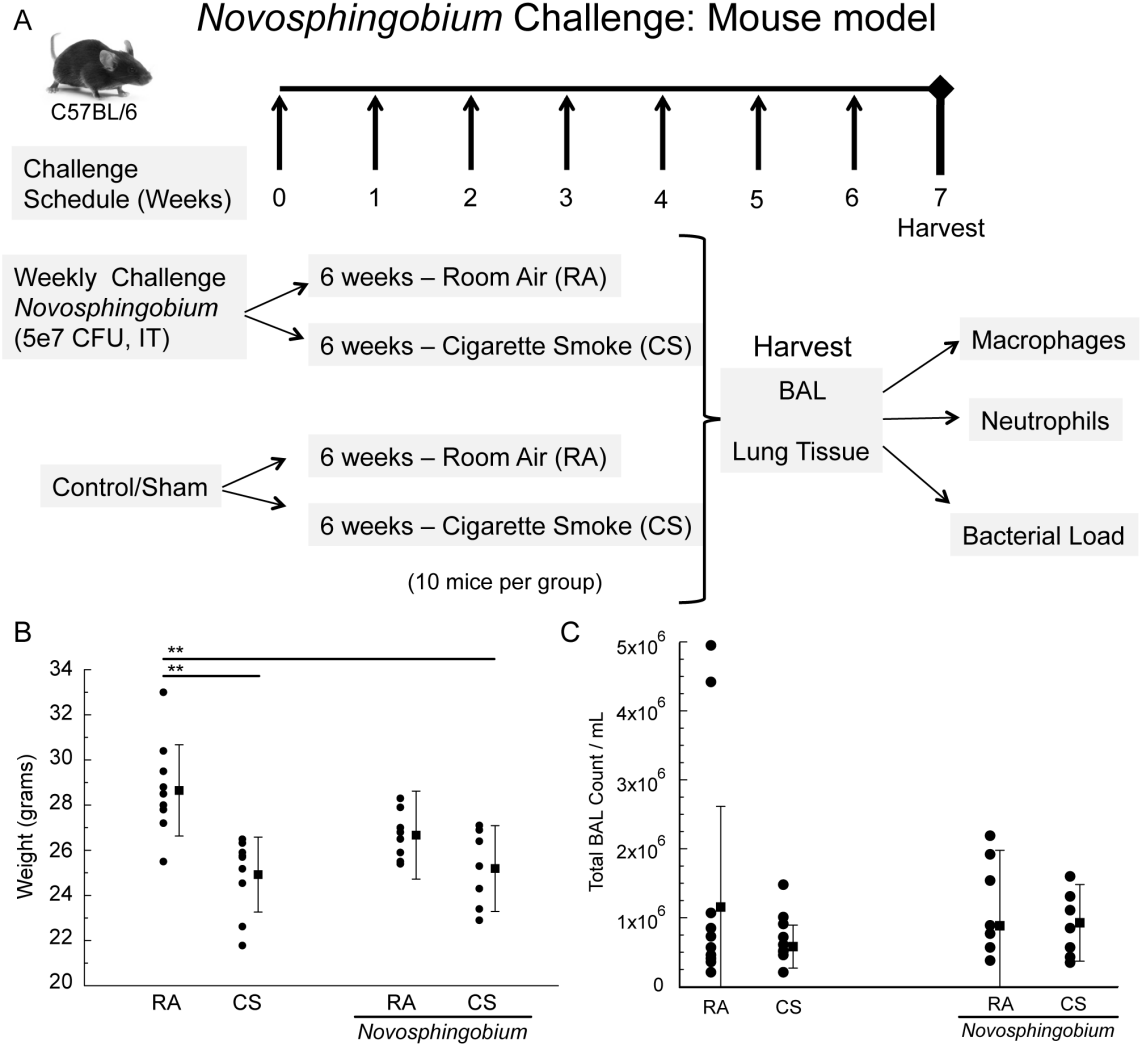
*Novosphingobium* mouse challenge model (A). C57BL/6 mice were challenged once every seven days with 5×10^7 ^CFU total of *Novosphingobium panipatense*. Mice were then exposed to room air conditions or to cigarette smoke from 3R4F reference cigarettes for six weeks. Control mice, challenged with sterile PBS, were exposed to room air or to cigarette smoke for six weeks. At the end of the six weeks of exposure, BAL and lung tissue were harvested, processed and analyzed for markers of inflammation and levels of bacteria. (B) Body weight of C57BL/6 mice after six weeks of weekly challenge with either 5×10^7 ^CFU total of *Novosphingobium panipatense* or sterile PBS. Mice were exposed to room air conditions or to cigarette smoke from 3R4F reference cigarettes during the six weeks of challenge. (C) Total BAL cell counts. Bronchoalveolar lavage (BAL) fluid was collected from mouse lungs by aspiration with phosphate buffered saline which was repeated three times. Total viable cell counts were determined in a hemocytometer using trypan blue exclusion. (One-way ANOVA test, **significant at p<0.01).

Given our findings with analysis of human tissues, we relied on the mouse model of alveolar inflammation and cell death due to cigarette smoke to further the understanding of the potential role of to *Novosphingobium* in COPD. A bacterial suspension containing 5×10^7^ cfu *N*. *panipatense* was administered intratracheally (IT) to mice 6–8 weeks of age. Following administration of the bacteria, the animals were exposed to either room air or cigarette smoke as described in the materials and methods section and outlined in Figure 3A.

### *Novosphingobium panipatense*-induced recruitment of tissue markers of inflammation in mouse Bronchoalveolar Lavage

Analysis of body weight demonstrated a significant decrease in body weight in cigarette smoke–exposed compared to room air control mice (24.9 g vs. 28.7 g, p<0.01, Figure 3B). Bacterial challenge with *N. panipatense* IT similarly resulted in total body weight loss in room air and significantly so in cigarette smoke–exposed mice compared with mice that did not receive bacterial challenge (25.2 g vs. 28.7 g, p<0.01).

### Bronchoalveolar Lavage total and differential cell counts

**Figure 4:**
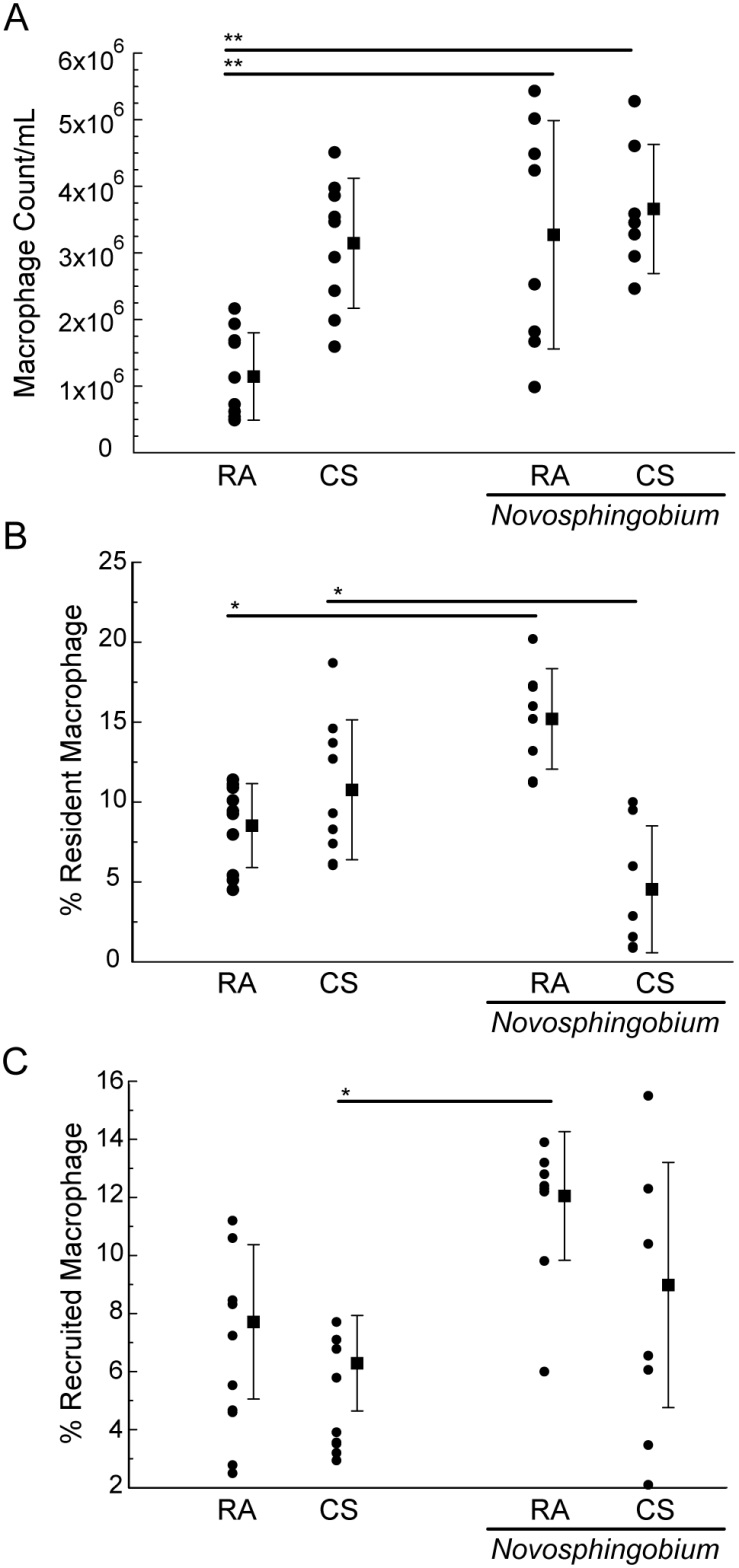
Macrophage cell counts. (A) Differential counts for macrophages were assessed ex vivo using cytospin smears of BAL samples. 150 µL of cell suspension was centrifuged in a Shandon Single Cytofunnel for 3 minutes. The slides were processed and stained using 3 Hema 3 step stain set and cells were scored. Results were expressed as total cell number/mL. (B) Macrophage Inflammatory responses assessed by flow cytometry on mouse lung tissue-recovered cells. For each mouse experimental group, 50,000–100,000 events were collected on an LSRII flow cytometer and analyzed for the presence of resident macrophages (CD11c+Siglec F+) (C) and recruited macrophages (CD11b+Siglec F−). (One-way ANOVA test, significant at *p<0.05, **p<0.01).

**Figure 5:**
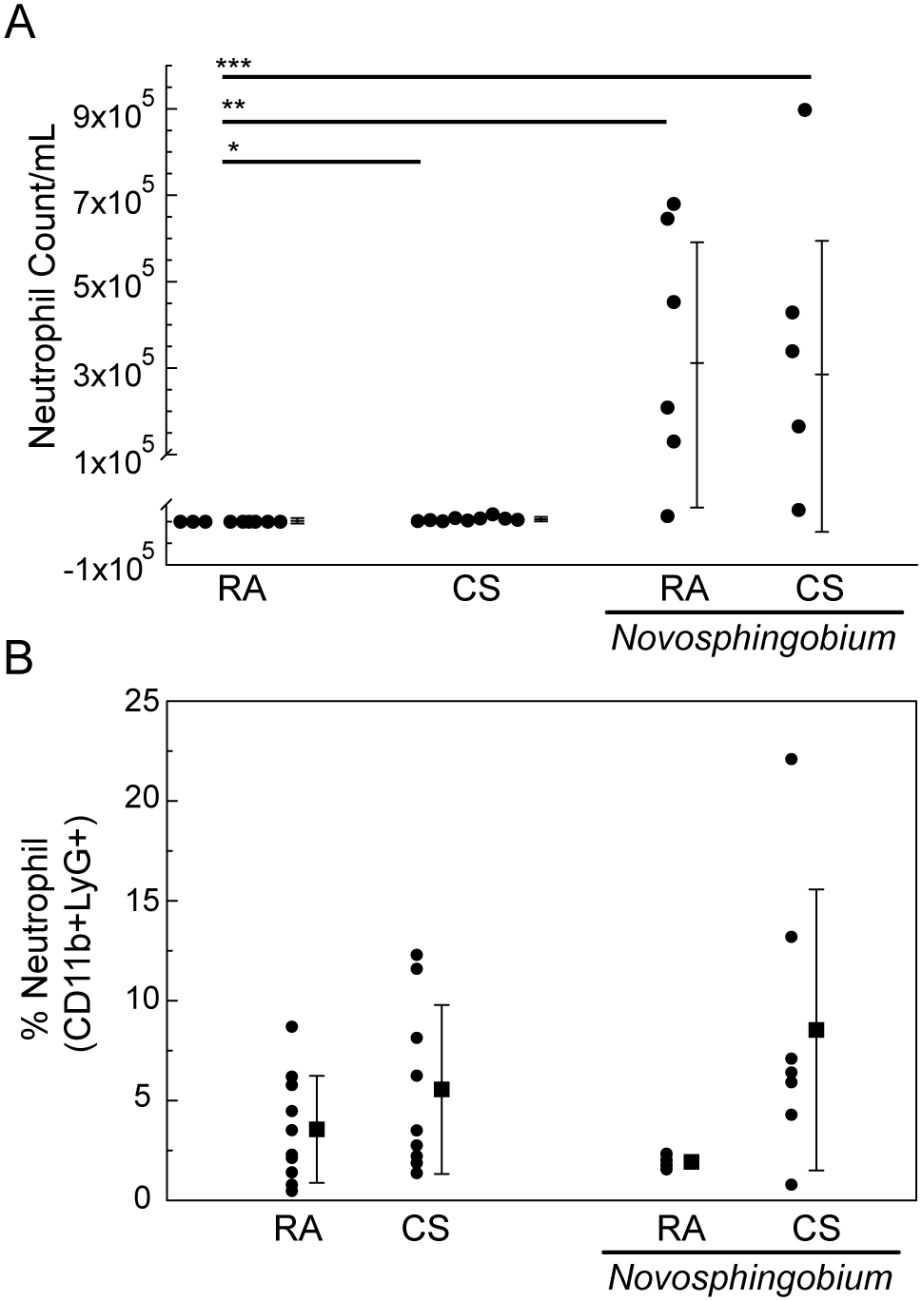
Neutrophil cell counts. (A) Differential counts for neutrophils were assessed ex vivo using cytospin smears of BAL samples. Results were expressed as total cell number/mL. (B) Neutrophil Inflammatory responses assessed ex vivo by flow cytometry on mouse lung tissue-recovered cells. For each mouse experimental group, 50,000–100,000 events were collected on an LSRII flow cytometer and analyzed for the presence of neutrophils (Ly6G+CD11b+). (One-way ANOVA test, significant at *p<0.05, **p<0.01 and ***p<0.001).

Inflammatory responses were assessed *ex vivo* by total and differential cell counts of cells retrieved by BAL. There were no significant differences in total BAL cell counts between control samples and samples from mice challenged with *N*. *panipatense* (Figure 3C). However, differential cell counts revealed increased inflammatory macrophage cell influx in cigarette exposed mice (3.16×10^6^ cells/mL, Figure 4A) compared to control mice treated to 6 weeks of room air (1.15×10^6^ cells/mL). Significant macrophage cell influx was also observed when mice were subjected to challenge *N. panipatense* and either exposed to the cigarette smoke or room air (3.27×10^6^ cells/mL, p<0.001; and 3.66×10^6^ cells/mL, p<0.001, respectively). Airway inflammation assessed by enumeration of neutrophils revealed significant increased neutrophil cell influx in cigarette exposed mice (5.95×10^3^ cells/mL) compared to control mice treated for 6 weeks with room air (2.00×10^3^ cells/mL, p<0.05). Neutrophil cell influx was also increased when mice were subjected to *N. panipatense* challenge and either exposed to the presence or absence of cigarette smoke (2.85×10^5^ cells/mL, p<0.05; and 3.12×10^5^ cells/mL, p<0.01, respectively) (Figure 5A).

### *Novosphingobium panipatense*-induced recruitment of markers of inflammation in mouse lung tissue-recovered cells

Inflammatory responses were assessed *ex vivo* by flow cytometry on mouse lung tissue-recovered cells. The proportion of resident macrophages was increased in cigarette exposed mice compared to control mice treated to 6 weeks of room air, though this observation did not reach significance (Figures 4B). However, mice challenged weekly with *N*. *panipatense* in the presence of room air, demonstrated a marked increase in the proportion of resident macrophages (CD11c+Siglec F+) in comparison to control mice treated to 6 weeks of room air (p<0.05). This observation was inverted when mice were subjected to *N*. *panipatense* challenge IT in the presence of cigarette smoke (Figures 4B). Cigarette smoke exposure appeared to alter the macrophage inflammatory response to the bacterial challenge, possibly indicating a shift of the inflammatory profile in the lung tissue, in that it decreased the numbers of resident macrophages, while not affecting the recruited population (Figures 4A and B). In six of the mice, there was an increase in the percent of neutrophils in samples from mice challenged with *N*. *panipatense*, however, there was variance in the data and it did not reach levels of significance (Figure 5B).

### Clearance of *Novosphingobium panipatense* from mouse lungs

**Figure 6:**
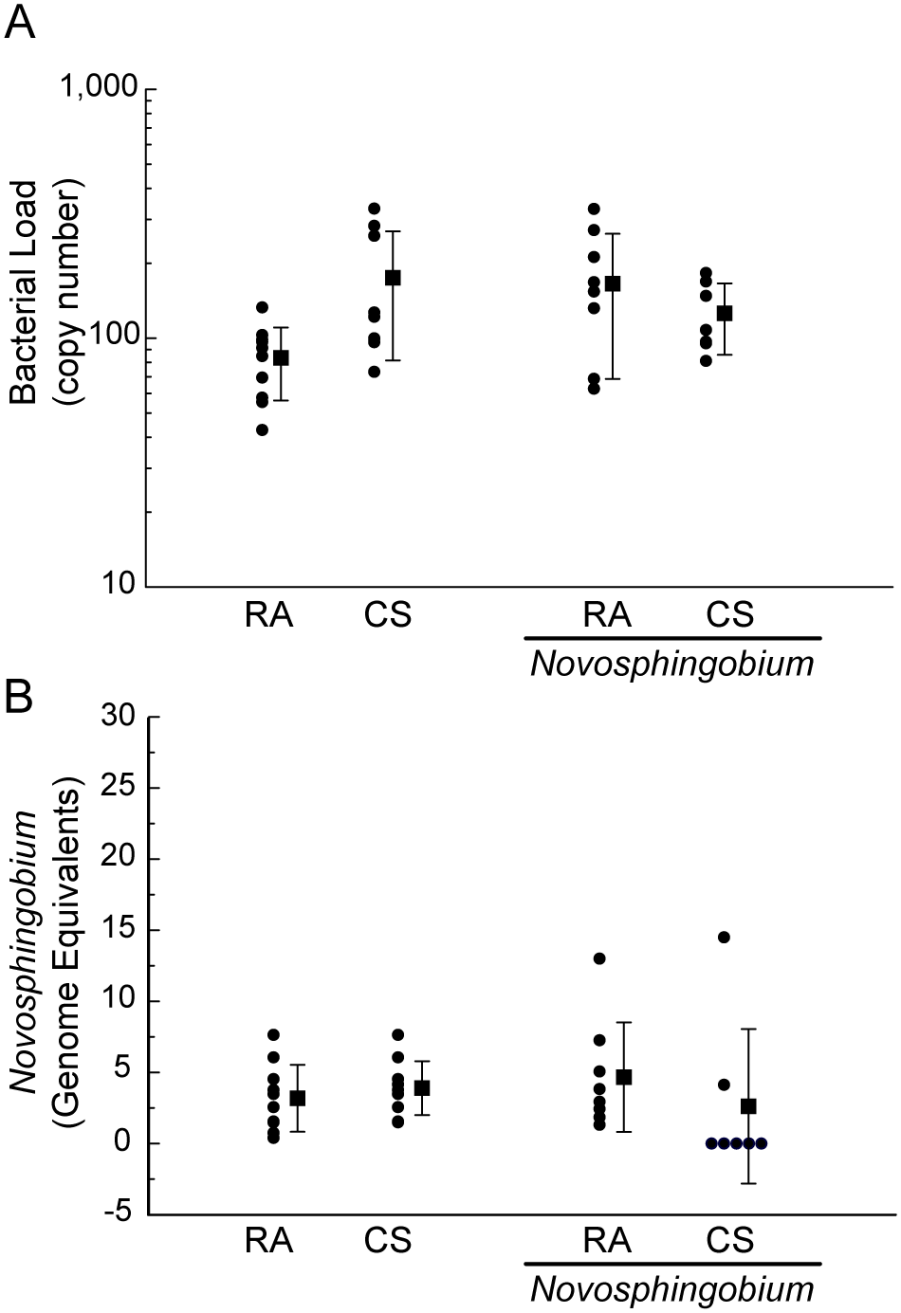
Bacterial detection in mouse lung tissue. (A) Evaluation of the presence and levels of total bacteria by qPCR on mouse lung tissue. DNA was extracted from mouse lung tissue and analyzed for levels of total bacteria in samples harvested six weeks post weekly IT challenge with either sterile PBS or with 5×10^7 ^CFU total of *Novosphingobium panipatense*. (B) Evaluation of the presence and levels of *Novosphingobium* by a two-step *Novosphingobium*-specific qPCR on mouse lung tissue. DNA was extracted from mouse lung tissue and analyzed for levels of *Novosphingobium* in samples harvested six weeks post weekly IT challenge with either sterile PBS or with 5×10^7 ^CFU total of *Novosphingobium panipatense*. The *Novosphingobium*-specific qPCR performed on these tissue samples resulted in undetectable levels of *Novosphingobium* in all tissue samples.

**Figure 7:**
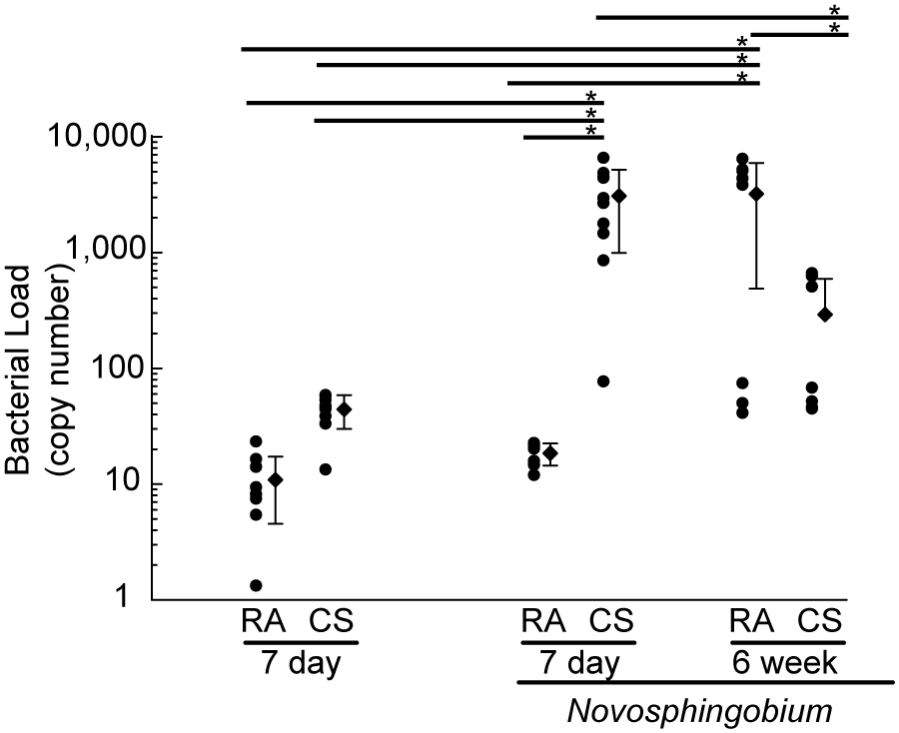
Evaluation of the presence and levels of total bacteria by qPCR on BAL. BAL samples were harvested from mice, seven days or six weeks post IT challenge with either sterile PBS or with 5×10^7 ^CFU total of *Novosphingobium panipatense*. BAL was analyzed by an all-bacteria qPCR. (One-way ANOVA test, significant at *p<0.05).

The lung is continuously exposed to bacteria in inhaled air. The normal sterility of the distal portions of the lung and the rapid decline in viable bacteria after infection or challenge, demonstrates the effectiveness of the pulmonary defense mechanisms. Inhaled bacteria disappear rapidly from the lungs of experimental animals and phagocytic cells on the surface of the alveoli and respiratory bronchioles are thought to play a major role in the mechanism of early and rapid uptake of bacteria and subsequent resistance to bacterial infection. Following IT challenge with *N. panipatense*, we sought to evaluate the presence and levels of bacteria by performing qPCR on BAL samples on a sub-set of mice, seven days post IT challenge and also on the mice that underwent 6 weeks of *N. panipatense* IT challenge (Figure 6A). All-bacteria qPCR was performed on BAL samples obtained seven days after bacterial IT challenge. The all-bacteria qPCR showed increased levels of bacteria in mice that were challenged IT and then subsequently exposed to cigarette smoke for the seven days following challenge compared to levels of bacteria in control groups (Figure 7). Similarly, after six weeks of bacterial IT challenge and exposure to either room air or cigarette smoke, increased levels of bacteria in the BAL (Figure 7), compared to BAL from seven smoke exposures in the presence and absence of IT challenge, as well as 7 day room air mice that were challenged IT, however, there was variance in the data and it did not reach levels of significance. The levels of total bacteria in tissue samples harvested after six weeks, however, revealed low (<400 copies/mL) levels of bacteria in all mouse groups (Figure 6A), with the lowest levels being seen in the six week room air control mice. The *Novosphingobium*-specific qPCR performed on these tissue samples similarly resulted in undetectable levels of *Novosphingobium* (Figure 6B). Significantly lower bacterial load was observed in mice that were exposed to smoke and challenged with *N. panipatense* for 6 weeks compared to mice either exposed to smoke and challenged with *N. panipatense* for 7 days or to mice exposed to room air and challenged with N. panipatense for 6 weeks. These mice appeared to clear the bacteria challenge during the 6-week interval (Figure 7).

## Discussion

Based on deep DNA sequencing, we estimated the number of bacterial species in lung tissues obtained from normal lungs and from diseased lungs by extrapolation based on amplified and sequenced 16S rRNA genes. Our results are consistent with prior studies, which that show that lungs of patients with severe COPD retained higher microbial diversity than disease control subjects [48]. However, we were surprised to obtain sequence data that revealed the presence of *Novosphingobium* spp. at high relative abundance (>2% relative abuncance) in both normal lungs and diseases lungs. Within diseased lungs, levels of *Novosphingobium* spp. were observed more frequently in samples from subjects with advanced COPD (GOLD stage 3 and 4, 18/27 samples) compared to samples from subjects either not at risk or with early-moderate COPD (GOLD stage 0, 1 and 2, 4/11 samples). Our results are in concordance with several other clinical studies that have reported the presence of lung microbiota in patients with COPD [12], [48]–[51]. Our data confirms prior studies that have reported *Novosphingobium* spp. sequences from lung tissue [12], [48], [52]. It is important to note that the high relative abundance of *Novosphingobium* spp. has not been highlighted in other settings.

To date, a clear connection between microbiome diversity and respiratory disease has not been well described. Moreover, no further attempts have been made previously to use deep bacterial sequencing to identify potentially pathogenic bacteria. In particular, the contribution and role of sphingomonads such as *Novosphingobium* in diseased and healthy lungs remains unclear. To address this goal, we developed a *Novosphingobium*-specific qPCR to quantify levels of *Novosphingobium* spp. in the human lung tissue. Quantitative PCR was concordant with prior sequence data and high levels of *Novosphingobium* were quantifiable in advanced COPD, but not from other COPD disease stages. The *Novosphingobium*-specific qPCR demonstrates the potential for sensitive and high throughput approaches to defining and quantifying specific bacteria.

The finding of abundant and frequent detection of *Novosphingobium* spp. in human samples was initially intriguing. Given that *Novosphingobium* spp. are α-Proteobacteria, common in soil and at liquid surfaces (i.e., water pools), we were concerned that it represented a procedural contaminant. The aggregate of evidence argues against this hypothesis. Firstly, we determined that the lung samples provided by the LTRC were collected from multiple academic sites, at different periods of time. Secondly, we also detected *Novosphingobium* DNA in lung tissue, which was processed (i.e., sectioned, aliquoted, frozen, and stored) at different sites. Thirdly, several internal controls involving the procedural steps of DNA extraction, PCR amplification, and sequencing also failed to reveal potential sources of contamination. Importantly, most of the microbiome studies to date aimed at samples obtained from patients with asthma and COPD detected *Novosphingobium* spp. in the bronchoalveolar, brushing, and lung tissue DNA [12], [48], [52], however at different abundance as that found in our studies.

Several pieces of evidence lead us to focus on *Novosphingobium* spp. Rather than containing lipopolysaccharide present in Gram negative bacteria, the family *Sphingomonadaceae* (which include the genera *Novosphingobium*, *Sphingomonas*, and *Sphyngopyxis*) [53], contains sphingolipids in their wall and are capable of metabolizing polyaromatic hydrocarbons [47], as present in tobacco leaves. *Novosphingobium* spp. have been found to be present in multiple environmental surfaces [54], as a contaminant in respiratory therapy items [55]. *Novosphingobium* spp. typically grows in nutrient agar at 30°C, which may explain why these bacteria were not detected in clinical studies of acute exacerbations in COPD. Infection or colonization of vertebrate hosts, including humans, can however occur at 37°C as documented of sporadic infection by *Sphingomonas* of humans [56]. A potentially important implication of a pathogenic role for *Novosphingobium* spp. in COPD is the association of *Novosphingobium aromaticivorans* in primary biliary cirrhosis, an autoimmune disease of the liver [46], in which the signature anti-antibody against the mitochondria pyruvate dehydrogenase complex also recognizes the bacterial enzyme ortholog [45]. Due to the increasing data that supports autoimmunity as a potential component to the exacerbation of COPD [31], there was compelling evidence to move forward and analyze these bacteria, as it may have effects on the lung system in addition to the liver. In this present study, we confirmed that COPD lung samples can be segregated as containing high or low *Novosphingobium* spp. burden.

We then used a sub-acute inflammation mouse model to mimic acute lung inflammation and to quantify innate immune cell sub-populations responding to *Novosphingobium* bacterial challenge IT, in the presence and the absence of cigarette smoke. BAL from C57BL/6 mice challenged IT with *Novosphingobium* spp. and exposed to cigarette smoke demonstrated increased levels of neutrophils and macrophages compared to room air controls. In addition, by flow cytometry, we determined the populations of specific inflammatory cells in mouse lung tissue-recovered cells. Compared with PBS- inoculated control animals at room air, mice challenged IT with live *N. panipatense* exhibited an increase in the numbers of neutrophils. The total number of resident and recruited macrophages was increased in the mouse lung tissue-recovered cells. *Novosphingobium* appears to induce an inflammatory response in mice that subsequently clears and/or controls the infection. The effect of *Novosphingobium* spp. on the inflammatory response, in conjunction with cigarette smoke, was unclear and requires further clarification. This limited response could be accounted by the inability to accurately model the chronic lung injury imposed by decades of cigarette smoking in an abbreviated time frame of 6 weeks in the mouse. Moreover, this model may not reproduce the immunologic and structural abnormalities seen in patients with the disease.

Longitudinal analysis in COPD patient populations may help to uncover the effects and contributions of these microbial populations in the lung. Clarifying the presence of specific microbiota in the lung may help to reveal under-recognized bacterial species that potentially are pathogenic or that may alter the microbiome during or by inducing, inflammation and airway destruction. Notwithstanding, this study does present with some limitations that include the absence of a primary species and human isolate of *Novosphingobium*. The dynamics of bacterial colonization in the lung, particularly during the different stages of disease, are not well understood; therefore recognition of the specific bacterial colonization in lung disease could lead to increased understanding of their roles in disease. Another limitation of the study may be the bacterial challenge dose that was administered to the mice [57]. Different bacterial doses of *Novosphingobium* spp. may have an effect on the immune response of the host and on the inflammatory response that results. The infectious dose for *Novosphingobium* spp. is unknown and likely differs depending upon the host. Finally, the use of a mouse model to reflect sub-acute COPD in humans may also be limiting.

The study presented here sought to investigate the role of *Novosphingobium* spp. in a sub-acute inflammation mouse model, and is one of few studies that have explored the functional effects of the microbiome in lung disease. We postulated that Novosphingobium in the presence of disease may have a different function and play a role in disease progression and as such, embarked on using an animal model, incorporating both cigarette smoke and bacterial challenge to verify the hypothesis that Novosphingobium would be increased in COPD, and also to determine a possible role of Novosphingobium in lung inflammation and disease. In this mild model of COPD, the immune response of the animals was sufficient to clear the organism even with repeated challenge. Novosphingobium may therefore unlikely be pathogenic, alternatively the mouse model may not be the most suitable host in which to determine the role of the Novosphingobium bacteria. It therefore remains unclear what long-term exposure would reveal in a more suitable host. We found Novosphingobium in control, smokers’ and diseased samples. Novosphingobium therefore appears to be part of a normal lung microbiome. However, it is conceivable that it may play pathogenic roles, notably in a diseased lung. The results of this analysis contribute to current knowledge and extend previous reports about the airway bacterial microbiome in the context of lung disease.
